# The joint impact on being overweight of self reported behaviours of eating quickly and eating until full : cross sectional survey

**DOI:** 10.1136/bmj.a2002

**Published:** 2008-10-21

**Authors:** Koutatsu Maruyama, Shinichi Sato, Tetsuya Ohira, Kenji Maeda, Hiroyuki Noda, Yoshimi Kubota, Setsuko Nishimura, Akihiko Kitamura, Masahiko Kiyama, Takeo Okada, Hironori Imano, Masakazu Nakamura, Yoshinori Ishikawa, Michinori Kurokawa, Satoshi Sasaki, Hiroyasu Iso

**Affiliations:** 1 Department of Social and Environmental Medicine, Graduate School of Medicine, Osaka University, Yamadaoka, 2-2 Suita-shi, Osaka, Japan 565-0871; 2 Osaka Medical Center for Health Science and Promotion, Osaka, Japan; 3 Chiba Prefectural Institute of Public Health, Chiba-City, Japan; 4 Harvard Center for Population and Development Studies, Harvard University, MA, USA; 5 Division of Health and Welfare, Osaka Prefecture, Japan; 6 Department of Social and Preventive Epidemiology, School of Public Health, University of Tokyo, Japan

## Abstract

**Objective** To examine whether eating until full or eating quickly or combinations of these eating behaviours are associated with being overweight.

**Design and participants** Cross sectional survey.

**Setting** Two communities in Japan.

**Participants** 3287 adults (1122 men, 2165 women) aged 30-69 who participated in surveys on cardiovascular risk from 2003 to 2006.

**Main outcome measures** Body mass index (overweight ≥25.0) and the dietary habits of eating until full (lifestyle questionnaire) and speed of eating (validated brief self administered questionnaire).

**Results** 571 (50.9%) men and 1265 (58.4%) women self reported eating until full, and 523 (45.6%) men and 785 (36.3%) women self reported eating quickly. For both sexes the highest age adjusted mean values for height, weight, body mass index, and total energy intake were in the eating until full and eating quickly group compared with the not eating until full and not eating quickly group. The multivariable adjusted odds ratio of being overweight for eating until full was 2.00 (95% confidence interval 1.53 to 2.62) for men and 1.92 (1.53 to 2.40) for women and for eating quickly was 1.84 (1.42 to 2.38) for men and 2.09 (1.69 to 2.59) for women. The multivariable odds ratio of being overweight with both eating behaviours compared with neither was 3.13 (2.20 to 4.45) for men and 3.21 (2.41 to 4.29) for women.

**Conclusion** Eating until full and eating quickly are associated with being overweight in Japanese men and women, and these eating behaviours combined may have a substantial impact on being overweight.

## Introduction

Obesity or being overweight is an important risk factor for lifestyle related diseases such as cancer, cardiovascular diseases, and diabetes.^1^ In recent years the prevalence of overweight and obesity has increased both worldwide and among Japanese men.^2^ Weight gain is caused by energy intake in excess of energy expenditure.^3^ ^4^ Eating quickly, gorging, and binge eating have been associated with total energy intake,^5^ ^6^ ^7^ and eating quickly and binge eating have been associated with satiety^8^ ^9^ and insulin resistance^10^ ^11^ All these eating behaviours may lead to being overweight or obese.^12^ ^13^ ^14^ In addition, the positive association of eating quickly with body mass index was observed independent of total energy intake.^15^ ^16^

Eating until full refers to eating a large quantity of food in one meal and is unrelated to eating disorders, whereas gorging is characterised by few meals but a large quantity consumed during one meal^5^ ^13^ and binge eating by the ingestion of abnormally large quantities of food many times a day.^17^

We examined whether eating until full and eating quickly are associated with being overweight in a population based sample of adults in Japan. We also examined the combined effect of eating until full and speed of eating on being overweight.

## Methods

We carried out a cross sectional study of 4140 adults (1496 men, 2644 women) aged 30 to 69 in two Japanese communities who participated in surveys on cardiovascular risk from 2003 to 2006 under the Japan health law. The surveys were carried out in Ikawa, a rural community in the north east of Japan, and Yao, a suburb in the south west.

Overall, 3650 (88.2%) participants responded to self administered questionnaires on diet history and 489 (12%) refused. Overweight and body mass index were similar between the two groups.

We excluded participants with a history of cardiovascular diseases (n=308), excessively high (>4000 kcal) or low (<500 kcal) total reported daily energy intake (n=20), and lacking data related to eating until full or speed of eating (n=35). The data for the remaining 3287 participants (1122 men, 2165 women) were used for the analyses.

### Measurements

To avoid measurement bias we used standardised methods to carry out the surveys, the details of which are described elsewhere.^18^ ^19^ We measured the participants’ height without footwear and weight in light clothing and calculated their body mass index (weight (kg)/(height (m)^2^). For the purposes of the analysis we considered a body mass index of 25.0 or more as indicating overweight. We also interviewed participants to ascertain data on smoking status, the number of cigarettes smoked daily, occupation, and the use of regular physical exercise for 15 minutes or more a week.

### Dietary assessments

We used a validated, self administered, brief questionnaire on diet history to assess the participants’ dietary habits during the previous month.^20^ ^21^ ^22^ The participants were asked whether they usually eat until full (yes or no) and speed of eating was self reported according to one of five qualitative categories: very slow, slow, medium, fast, and very fast. Owing to small numbers of participants in the very fast category we combined the very fast and fast categories into the category for eating quickly. We validated the self reported speed of eating as used previously.^15^ Self reported speed of eating showed a high level of agreement with speed of eating as reported by a friend: the percentages of exact and adjunct categories of answers (for example, very fast and fast were regarded as agreed) were 46% and 47%, respectively.^15^ After we had combined the categories for very fast and fast and also combined the categories for medium, slow, and very slow, the percentage of agreement was reasonably good (75.3%), with a moderate κ statistics (0.35). We tested the repeatability for self reporting eating until full and eating quickly (very fast and fast combined) by repeating the questionnaire survey after one year in a subsample of the participants (1062 men, 1816 women). The κ statistics for eating until full were 0.60 in men and 0.63 in women, and for eating quickly were 0.63 in men and 0.67 in women.

### Statistical analysis

We calculated age adjusted mean values for participants’ characteristics using analysis of covariance and age adjusted proportions by using logistic regression according to the combination of eating until full and eating quickly.

We calculated odds ratios and 95% confidence intervals by using the logistic regression model for age adjusted odds ratios and multivariable adjusted odds ratios. The multivariable adjustment included age (years), total energy intake (kcal/day), total fibre and alcohol intake (g/day), smoking status (non-smoker; former smoker; and 1-20, 21-40, and ≥41 cigarettes consumed daily), occupation (desk worker, service business, manual labour, unemployed), regular physical activity (yes or no), and survey area (Ikawa or Yao).

We also tried to determine whether there was a supra-additive association (additive interaction) between eating until full and eating quickly. The relative excess risk due to interaction is the excess risk as a result of joint exposure. In terms of the model coefficients, the relative excess risk due to interaction is calculated as exponent(β_1_+β_2_+β_3_)−exponent(β_1_)−exponent(β_2_)+1 where β1, β2, and β3 are the coefficients from the model for specified levels of eating until full and eating quickly, as well as their interaction. Thus, the relative excess risk due to interaction equals the odds ratio(eating until full+eating quickly)−odds ratio(eating until full)−odds ratio(eating quickly)+1. We divided the statistic by the square root of its estimated variance to test the hypothesis that the relative excess risk due to interaction equalled zero with a z test (normal distribution) approximation.^23^ ^24^ This increase in excess risk due to interaction of the two categories as a percentage of the increase in risk as a result of joint exposure (relative excess risk due to interaction percentage) is then expressed as (relative excess risk due to interaction/[odds ratio(eating until full+eating quickly)−1])×100. The percentage relative excess risk due to interaction is defined as the proportion of disease burden caused by two factors that can be attributed to their interaction. We also calculated the attributable proportion due to interaction=relative excess risk due to interaction/odds ratio(eating until full+eating quickly)×100—that is, the proportion of overweight among those both eating until full and eating quickly that is attributable to interaction.

Probability values for statistical tests were two tailed and we regarded P<0.05 as statistically significant. We used the SAS statistical package version 9.1 for the analyses.

## Results

Table 1 shows the baseline characteristics of the participants. The mean (standard deviation) age of participants was 55.3 (10.7) for men and 52.4 (11.1) for women, with 379 (33.8%) men and 472 (21.8%) women being overweight. Overall, 571 (50.9%) men and 1265 (58.4%) women reported eating until full and 523 (45.6%) men and 785 (36.3%) women reported eating quickly (very fast and fast categories combined). For both sexes the eating until full and eating quickly group had the highest age adjusted mean values for height, weight, body mass index, and total energy intake than did the group with neither of these eating behaviours.

**Table 1 tbl1:** Characteristics of participants according to combination of eating until full and eating quickly. Values are means (standard errors) unless stated otherwise

Characteristics	Not eating until full, not eating quickly	Eating until full, not eating quickly	Not eating until full, eating quickly	Eating until full, eating quickly	Total
Men:	n=352	n=258	n=199	n=313	n=1122
Mean (SD) age (years)	58.1 (10.0)	54.8 (10.3)	57.0 (9.7)	51.4 (11.3)	55.3 (10.7)
Height (cm)	164.8 (0.3)	165.8 (0.4)	165.9 (0.4)	166.6 (0.3)	165.7 (0.2)
Weight (kg)	63.1 (0.5)	66.8 (0.6)	64.9 (0.7)	69.6 (0.5)	66.1 (0.3)
Body mass index (kg/m^2^)	23.2 (0.2)	24.3 (0.2)	23.6 (0.2)	25.0 (0.2)	24.0 (0.1)
Total energy intake (kcal)	2190 (30)	2296 (35)	2143 (40)	2296 (32)	2236 (17)
Total protein intake (% energy)	14.1 (0.1)	13.8 (0.2)	14.2 (0.2)	13.7 (0.1)	13.9 (0.1)
Total fat intake (% energy)	23.2 (0.3)	22.6 (0.3)	23.0 (0.4)	22.7 (0.3)	22.9 (0.2)
Carbohydrate intake (% energy)	53.2 (0.4)	53.2 (0.5)	53.2 (0.6)	53.8 (0.5)	53.4 (0.2)
Total dietary fibre intake (g/1000 kcal)	5.5 (0.1)	5.4 (0.1)	5.6 (0.1)	5.4 (0.1)	5.5 (0.0)
Alcohol intake (% energy)	7.7 (0.4)	8.6 (0.5)	7.7 (0.6)	7.9 (0.5)	8.0 (0.2)
Overweight (%)	23.1	33.1	30.2	48.7	33.8
Current smoker (%)	51.0	46.7	52.6	40.6	47.4
Desk worker (%)	10.5	11.8	9.4	10.3	10.5
Regular physical activity (%)	35.6	32.7	36.5	36.3	35.3
Women:	n=668	n=712	n=232	n=553	n=2165
Mean (SD) age (years)	54.6 (11.0)	51.2 (11.0)	53.2 (11.0)	50.9 (10.9)	52.4 (11.1)
Height (cm)	153.5 (0.2)	153.9 (0.2)	154.1 (0.3)	154.7 (0.2)	154.0 (0.1)
Weight (kg)	51.6 (0.3)	53.7 (0.3)	53.4 (0.5)	57.5 (0.3)	54.0 (0.2)
Body mass index (kg/m^2^)	21.9 (0.1)	22.7 (0.1)	22.5 (0.2)	24.0 (0.1)	22.8 (0.1)
Total energy intake (kcal)	1693 (17)	1812 (17)	1719 (29)	1840 (19)	1773 (10)
Total protein intake (% energy)	15.7 (0.1)	15.2 (0.1)	15.4 (0.2)	15.4 (0.1)	15.4 (0.1)
Total fat intake (% energy)	27.6 (0.2)	27.0 (0.2)	27.5 (0.3)	27.3 (0.2)	27.3 (0.1)
Carbohydrate intake (% energy)	53.9 (0.3)	55.0 (0.3)	54.7 (0.4)	55.0 (0.3)	54.6 (0.1)
Total dietary fibre intake (g/1000 kcal)	6.9 (0.1)	6.7 (0.1)	7.1 (0.1)	6.8 (0.1)	6.8 (0.0)
Alcohol intake (% energy)	1.4 (0.2)	1.4 (0.1)	1.1 (0.3)	1.0 (0.2)	1.3 (0.1)
Overweight (%)	14.0	19.9	19.9	34.3	21.8
Current smoker (%)	10.5	8.5	11.2	10.7	10.0
Desk worker (%)	4.0	5.5	5.8	6.2	5.2
Regular physical activity (%)	39.0	35.5	41.7	38.9	38.1

All values are adjusted for age except for number of overweight, current smoker, desk worker, and engaging in regular physical activity.

The eating until full group had higher age adjusted odds ratios for overweight than the not eating until full group for both men and women. The odds ratios were not changed substantially by further adjustment for intake of total energy, total fibre, and alcohol; smoking status; physical activity; and survey area: 2.00 (95% confidence interval 1.53 to 2.62) for men and 1.92 (1.53 to 2.40) for women. The eating quickly group had higher age adjusted odds ratios for overweight than did the not eating quickly group for both sexes. The multivariable adjusted odds ratios for overweight for the eating quickly group was 1.84 (1.42 to 2.38) for men and 2.09 (1.69 to 2.59) for women (table 2).

**Table 2 tbl2:** Age adjusted and multivariable adjusted odds ratios and 95% confidence intervals for overweight according to eating until full and eating quickly

Variable	Eating until full	Eating quickly
Men (n=1122):	n=571	n=512
No (%) overweight	234 (41.0)	210 (41.0)
Age adjusted odds ratio	2.04 (1.57 to 2.64)	1.85 (1.44 to 2.38)
Multivariable odds ratio*	2.00 (1.53 to 2.62)	1.84 (1.42 to 2.38)
Women (n=2165):	n=1265	n=785
No (%) overweight	324 (25.6)	233 (29.7)
Age adjusted odds ratio	1.93 (1.54 to 2.40)	2.11 (1.71 to 2.60)
Multivariable odds ratio*	1.92 (1.53 to 2.40)	2.09 (1.69 to 2.59)

*Adjusted for age; smoking status; regular physical activity; occupation; intake of total energy, total dietary fibre, and alcohol; and survey area.

The multivariable adjusted odds ratio for overweight for the eating until full and eating quickly group compared with the group with neither of these eating behaviours was 3.13 (2.20 to 4.45) for men and 3.21 (2.41 to 4.29) for women (table 3). On the basis of the multivariable adjusted model, the relative excess risk due to interaction for men was 1.10, indicating an excess burden of being overweight of 51.6% (percentage relative excess risk due to interaction, P<0.05) for eating until full and eating quickly, and for women was 1.27, indicating an excess burden of being overweight of 57.4% (percentage relative excess risk due to interaction, P<0.01; table 3). The attributable proportion due to interaction was 35.1% for men and 39.6% for women.

**Table 3 tbl3:** Age adjusted and multivariable adjusted odds ratios and 95% confidence intervals for overweight according to combinations of eating until full and eating quickly

Variable	Not eating until full, not eating quickly	Eating until full, not eating quickly	Not eating until full, eating quickly	Eating until full, eating quickly	RERI (RERI%)*
Men (n=1122):	n=352	n=258	n=199	n=313	
No (%) overweight	84 (23.8)	85 (32.9)	61 (30.7)	149 (47.6)	—
Age adjusted odds ratio	1.00	1.64 (1.14 to 2.35)	1.43 (0.97 to 2.11)	3.17 (2.25 to 4.47)	1.10 (50.7)
Multivariable odds ratio†	1.00	1.61 (1.11 to 2.32)	1.42 (0.96 to 2.11)	3.13 (2.20 to 4.45)	1.10 (51.6)
Women (n=2165):	n=668	n=712	n=232	n=553	
No (%) overweight	100 (15.0)	138 (19.4)	47 (20.3)	186 (33.6)	—
Age adjusted odds ratio	1.00	1.50 (1.13 to 2.00)	1.51 (1.02 to 2.22)	3.23 (2.44 to 4.28)	1.22 (54.9)
Multivariable odds ratio†	1.00	1.48 (1.10 to 1.98)	1.47 (0.99 to 2.17)	3.21 (2.41 to 4.29)	1.27 (57.4)

RERI=relative excess risk due to interaction.

*P<0.05 (z test).

†Adjusted for age; smoking status; regular physical activity; occupation; intake of total energy, total dietary fibre, and alcohol; and survey area.

## Discussion

Eating until full and eating quickly were significantly associated with overweight in Japanese men and women after adjustment for total energy intake and other potential confounding factors. The combination of the two eating behaviours had a supra-additive effect (additive interaction) on being overweight.

Eating quickly is positively associated with body mass index and increased body weight among Japanese^15^ ^16^ and Western populations.^12^ The questionnaire for evaluation of speed of eating used in the present study was the same as the one used in previous studies,^15^ ^16^ and the findings of the present and previous studies showed essentially the same trends. One study examined associations between the speed of eating and body mass index in Japanese women aged 18; the speed of eating (very slow, slow, medium, fast, and very fast) was found to be significantly and positively associated with body mass index.^15^ Another study also examined associations between the speed of eating and body mass index but in Japanese men and women aged 35-69 years.^16^ Furthermore, the speed of eating was positively associated with the homeostasis model assessment of insulin resistance for middle aged Japanese men and women without diabetes, especially for those who were not obese.^10^ Speed of eating was significantly and positively correlated with total energy intake, but the odds ratio for overweight did not change substantially after adjustment for total energy intake and other confounding variables. Therefore the effect of speed of eating may be unrelated to that of total energy intake.

One study investigated whether gorging was associated with overweight or obesity, but the epidemiological evidence was at best weak.^13^ Moreover, the present study observed that the combination of eating until full and eating quickly was strongly associated with being overweight.

The strength of our study is that we analysed the association of eating behaviour patterns with overweight using population based data for a large number of participants. The study does, however, have several potential limitations. Firstly, eating patterns were self reported and we did not determine the validity for self reporting of eating until full. The participants who reported eating until full, however, had higher total energy intake than the other participants, including those who reported gorging^5^ and binge eating, which supports the validity of the questionnaire.^7^ Secondly, we assessed eating behaviours as simplistic dichotomous outcomes. The validity and reproducibility of eating quickly and the reproducibility of eating until full were, however, reasonably good, and these eating behaviours as simplistic dichotomous outcomes were significantly associated with being overweight. Thirdly, we cannot deny the possibility that other potential confounding factors, such as educational history, may have had an effect on the observed associations. Fourthly, the cross sectional nature of the study indicates that the observed association between these eating behaviours and overweight does not necessarily indicate causality. It is unlikely, however, that people who are obese then change their eating habits. A cohort study of firefighters over seven years showed that eating quickly was associated with weight gain.^12^

In conclusion, eating until full and eating quickly were associated with being overweight in Japanese men and women, and the combination of the two eating behaviours may have a substantial impact on being overweight. As it is difficult to estimate these causal effects in a cross sectional study, prospective cohort and intervention studies will be needed to validate these associations between eating behaviour patterns and being overweight.

**What is already known on this topic**

Eating quickly, independent of total energy intake and other confounders, is associated with overweight

**What this study adds**

Both eating quickly and eating until full were associated with being overweight, independent of total energy intake and other confounders
These eating behaviours combined may have a substantial impact on being overweight

## Notes

We thank for their cooperation with this study the municipal authorities, officers, and physicians of Ikawa town, Yao city, and Osaka prefecture, and their colleagues in Osaka Medical Center for Health Science and Promotion.

Ethical approval: Osaka Medical Center for Health Science and Promotion research ethics committee.

Provenance and peer review: Not commissioned; externally peer reviewed.

## Notes

**Cite this as: ***BMJ* 2008;337:a2002
